# In vitro antiviral activity of fifteen plant extracts against avian infectious bronchitis virus

**DOI:** 10.1186/s12917-019-1925-6

**Published:** 2019-05-29

**Authors:** Raimundas Lelešius, Agneta Karpovaitė, Rūta Mickienė, Tomas Drevinskas, Nicola Tiso, Ona Ragažinskienė, Loreta Kubilienė, Audrius Maruška, Algirdas Šalomskas

**Affiliations:** 1 Institute of Microbiology and Virology, Veterinary Faculty, Lithuanian University of Health Sciences, Kaunas, Lithuania; 2 Department of Veterinary Pathobiology, Veterinary Faculty, Lithuanian University of Health Sciences, Kaunas, Lithuania; 3 Instrumental Analysis Open Access Centre, Vytautas Magnus University, Kaunas, Lithuania; 4 Sector of Medicinal Plants, Kaunas Botanical Garden of Vytautas Magnus University, Kaunas, Lithuania; 5 Faculty of Pharmacy, Lithuanian University of Health Sciences, Kaunas, Lithuania

**Keywords:** Avian infectious bronchitis, Plant extracts, Antiviral activity

## Abstract

**Background:**

Avian infectious bronchitis (IB) is a disease that can result in huge economic losses in the poultry industry. The high level of mutations of the IB virus (IBV) leads to the emergence of new serotypes and genotypes, and limits the efficacy of routine prevention. Medicinal plants, or substances derived from them, are being tested as options in the prevention of infectious diseases such as IB in many countries.

The objective of this study was to investigate extracts of 15 selected medicinal plants for anti-IBV activity.

**Results:**

Extracts of *S. montana*, *O. vulgare*, *M. piperita*, *M. officinalis*, *T. vulgaris*, *H. officinalis*, *S. officinalis* and *D. canadense* showed anti-IBV activity prior to and during infection, while *S. montana* showed activity prior to and after infection. *M. piperita*, *O. vulgare* and *T. vulgaris* extracts had > 60 SI. In further studies no virus plaques (plaque reduction rate 100%) or cytopathogenic effect (decrease of TCID_50_ from 2.0 to 5.0 log_10_) were detected after IBV treatment with extracts of *M. piperita*, *D. canadense* and *T. vulgaris* at concentrations of extracts ≥0.25 cytotoxic concentration (CC_50_) (*P* < 0.05). Both PFU number and TCID_50_ increased after the use of *M. piperita*, *D. canadense*, *T. vulgaris* and *M. officinalis* extracts, the concentrations of which were 0.125 CC_50_ and 0.25 CC_50_ (*P* < 0.05). Real-time PCR detected IBV RNA after treatment with all plant extracts using concentrations of 1:2 CC_50_, 1:4 CC_50_ and 1:8 CC_50_. Delta cycle threshold (Ct) values decreased significantly comparing Ct values of 1:2 CC_50_ and 1:8 CC_50_ dilutions (*P* < 0.05).

**Conclusions:**

Many extracts of plants acted against IBV prior to and during infection, but the most effective were those of *M. piperita*, *T. vulgaris* and *D. canadense* .

## Background

IB is a highly contagious respiratory and occasionally urogenital disease in chickens [1]. IBV affects the upper respiratory tract and reduces egg production [2]. It is a coronavirus that belongs to the *Coronaviridae* family. IBV is an enveloped virus with a single-stranded positive-sense linear RNA molecule (approximately 27.6 kb in size) [3].

IB has a wide geographical distribution and is diagnosed worldwide [1]. IB outbreaks continuously and results in economic losses in the poultry industry. So far vaccination using inactivated or live vaccines [4] is regarded as the main method of prevention, but it is not having the desired effect [5–7]. The high level of mutations of IBV [8] leads to the emergence of new serotypes and genotypes, and limits the efficacy of routine prevention.

Biological products derived from plants are used in medicine for different pharmacological reasons, including the treatment of infectious and non-infectious diseases [9, 10]. This class of antimicrobial plants is acknowledged and well investigated, and classes of active compounds have already been identified [11, 12]. The investigation of antiviral substances derived from plants is insufficient in comparison with the investigation of antimicrobial properties. Fortunately, several experiments have shown that plants have positive antiviral activity in vitro and in vivo [13]. However, the same plants can have different antiviral activity against RNA or DNA viruses, either enveloped or non-enveloped, and even against different types or strains of a virus [14, 15].

A number of scientific publications have encouraged the use of polyphenolic compounds in the treatment and prophylaxis of chronic diseases [16]. The mixture of the geometric isomers and enantiomers of rosmarinic acid is accumulating in the families *Lamiaceae* Lindl, *Asteraceae* Bercht. & J.Presl [17, 18]. Most large quantities of rosmarinic acid have been determined in the genus of plants such as *Salvia* L., *Perilla L. Melissa* L. and *Echinacea* Moench. Rosmarinic acid has antioxidative, anti-inflammatory, antimutagenic, antibacterial and antiviral effects against the herpes simplex virus [19].

The *Desmodium canadense* herb contains flavonoids such as apigenin, apigenin-7-O-glucoside, luteolin, rutin, 2-vicenin, vitexin, isovitexin, vitexin rhamnoside, orientin, homoorientin, quercetin, hyperoside, astragalin and kaempherol [20]. In addition, it also contains saponins and phenolic acids (chlorogenic acid, vanillic, 4- hydroxycinnamic, ferulic and caffeic). The *Desmodium* herb exhibits antioxidant, antibacterial, anti-inflammatory, hepatoprotective, diuretic and analgesic activity [20]. C-glycosides of flavonoids are known to exhibit antioxidant, hepatoprotective, anti-inflammatory and antiviral effects [21]. The plants in this study were chosen for their medical, antibacterial and antiviral properties. Ethanol extracts of medicinal plants belonging to the families *Lamiaceae* (winter savory, perilla, blue giant hyssop, oregano, peppermint, lemon balm, thyme, hyssop, catnip and sage), *Asteraceae* (chamomile and purple coneflower), *Geraniaceae* (rock crane’s-bill), *Apiaceae* (garden angelica), and *Fabaceae* (showy tick trefoil) were prepared. The majority of plants used for the preparation of extracts in this study belong to one of the famous medicinal aromatic plant families *Lamiaceae*. The medicinal plants from this family have long been used in traditional medicine worldwide.

Many investigations of plant extracts have been performed with different coronaviruses. The main targets were proteins involved in coronaviral replication, proteases and ion channel conductance [22]. Only a few investigations have been performed to test the anti-IBV activity of plant extracts. Several studies have found that the plant preparations inhibited IBV replication in vivo and vitro. *Sambucus nigra, Houttuynia cordata, Alium sativum* and *Astragalus mongholicus* inhibited IBV replication [23–26]. The ethanol extract of *Sambucus nigra* inhibited IBV replication and reduced virus titres prior to infection [24], as did *Houttuynia cordata* essential oil mixed with an aqueous solution of sodium chloride solution [25]. It is suggested that the effect of extracts of, *Alium sativum, Houttuynia cordata* and *Sambucus nigra* can be associated with direct inactivation of envelope structures of a virus, which are necessary for adsorption to or entry into host cells, or might dissolute the IBV envelope. Compounds that have a virucidal effect work like a disinfectant and do not require replication to inactivate the virus [15]. The mechanism of action of *Astragalus* polysaccharides has not been explained.

Medicinal plants or substances derived from them are being tested as a tool for preventing infectious diseases such as IB in many countries, but the anti-IBV viral properties of the selected plants have not so far been tested. The objective of this study was to investigate extracts of 15 selected medicinal plants for anti-IBV activity.

## Results

### Cytotoxicity of plant extracts

All the extracts were more cytotoxic (*P* < 0.05) than the ethanol control (7.7 μl). *A. foeniculum* showed the highest cytotoxic concentration (0.062 μg) and *P. frutescens* (0.77 μg) showed the lowest one.

### Antiviral effect against IBV

According to the results of the antiviral effect assay, eight extracts were selected for determination of the virucidal effect. The selected extracts of *S. montana, O. vulgare*, *M. piperita, M. officinalis, T. vulgaris, H. officinalis, S. officinalis* and *D. canadense* showed anti-IBV activity in two of the four methods. All eight extracts showed an antiviral effect prior to infection (method 1). Furthermore, seven of these showed antiviral activity during infection (method 2), while only the extract of *S. montana* showed anti-IBV activity after infection (method 4). *P. frutescens*, *N. cataria*, *E. purpurea*, *Ch. nobile* and *A. foeniculum* showed an antiviral effect only in the first method, while *G. macrorrhizum* and *A. archangelica* did not show an antiviral effect in any method (Table 1).

**Table 1:** Antiviral effect of plant extracts

No.	Latin name (family)	Part	Antiviral effect			
			Virus pre-treatment with extract			Cell pre-treatment prior to infection
			prior to infection	during infection	after infection	
1.	*Satureja montana*	herb	+	–	+	–
2.	*Chamaemelum nobile*	herb	+	–	–	–
3.	*Perilla frutescens*	herb	+	–	–	–
4.	*Agastache foeniculum*	herb	+	–	–	–
5.	*Origanum vulgare*	herb	+	+	–	–
6.	*Mentha piperita*	herb	+	+	–	–
7.	*Geranium macrorrhizum*	herb	–	–	–	–
8.	*Melissa officinalis*	herb	+	+	–	–
9.	*Angelica archangelica*	leaves	–	–	–	–
10.		roots	–	–	–	–
11.	*Thymus vulgaris*	herb	+	+	–	–
12.	*Hyssopus officinalis*	herb	+	+	–	–
13.	*Nepeta cataria*	herb	+	–	–	–
14.	*Echinacea purpurea*	herb	+	–	–	–
15.	*Salvia officinalis*	herb	+	+	–	–
16.	*Desmodium canadense*	herb	+	+	–	–

The above-mentioned eight plant extracts demonstrating anti-IBV activity were selected for further investigation. The 50% effective concentration (EC_50_) was determined in cells grown for 4 days (prior to infection). The EC_50_ values of extracts of *S. montana, O. vulgare*, *M. piperita, M. officinalis, T. vulgaris, H. officinalis, S. officinalis* and *D. canadense* were between 0.003 and 0.076 μg, however *S. officinalis* appeared effective at the lowest concentration (0.003 μg) (Table 2). SI of *M. piperita*, *O. vulgare*, and *T. vulgaris* extracts were 67.5, 65.0 and 63.1 respectively.

**Table 2:** Anti-IBV activity of some plant extracts in Vero cell cultures

No.	Plant extract	CC_50_ (μg)^a^	*EC*_*50*_ (μg)^b^	*SI*
1.	*Satureja montana*	0.75	0.044	17.0
5.	*Origanum vulgare*	0.52	0.008	65.0
6.	*Mentha piperita*	0.27	0.004	67.5
8.	*Melissa officinalis*	0.59	0.015	39.3
11.	*Thymus vulgaris*	0.63	0.010	63.1
12.	*Hyssopus officinalis*	0.64	0.076	8.4
15.	*Salvia officinalis*	0.11	0.003	36.7
16.	*Desmodium canadense*	0.29	0.017	17.1

^a^The assay for determination of CC_50_ was performed in octuplicate for each extract

^b^The experiments for determination of EC_50_ were repeated independently twice, and a mean is presented

### The anti-IBV activity prior to infection in Vero cells

The inhibitory effect of extracts against IBV prior to infection in Vero cells was measured by virus plaque and TCID_50_ assays. The selected plant extracts had a virucidal effect and inhibited viral replication compared to the virus control (*P* < 0.05). No virus plaques or CPE were detected after IBV treatment with extracts, whose concentrations were equivalent to 1 and 0.5 CC_50_ (Table 3).

**Table 3:** Virucidal effect and virus yield reduction

No.	Plant extract	Concentrations of plant extracts equivalent to CC_50_	PFU number (mean ± SD), log_10_	PFU reduction rate, %	TCID_50_ (mean ± SD), log_10_
1.	*S. montana*	1	0	100	0
		0.5	0	100	0
		0.25	0.97 ± 0.19	78.0	0.75 ± 0.64 ^a^
		0.125	1.03 ± 0.11	76.6	1.83 ± 0.31 ^c x^
5.	*O. vulgare*	1	0	100	0
		0.5	0	100	0
		0.25	1.08 ± 0.00	75.4	1.00 ± 0.94 ^a^
		0.125	1.40 ± 0.32	68.2	2.48 ± 0.23 ^d x^
6.	*M. piperita*	1	0	100	0
		0.5	0	100	0
		0.25	0	100	0^a^
		0.125	1.48 ± 0.05 ^x^	66.4	2.63 ± 0.13 ^d x^
8.	*M. officinalis*	1	0	100	0
		0.5	0	100	0
		0.25	0.15 ± 0.21	96.6	0.56 ± 0.44 ^a^
		0.125	1.72 ± 0.01 ^x^	60.9	3.38 ± 0.21 ^e x^
11.	*T. vulgaris*	1	0	100	0
		0.5	0	100	0
		0.25	0	100	0
		0.125	1.70 ± 0.01 ^x^	61.3	3.25 ± 0.22 ^ex^
12.	*H. officinalis*	1	0	100	0
		0.5	0	100	0
		0.25	1.33 ± 0.01	69.8	3.00 ± 0.29 ^b^
		0.125	2.00 ± 0.03 ^x^	54.5	3.35 ± 0.29 ^e^
15.	*S. officinalis*	1	0	100	0
		0.5	0	100	0
		0.25	2.00 ± 0.00	54.5	3.25 ± 0.25 ^b^
		0.125	2.19 ± 0.03 ^x^	50.2	3.45 ± 0.21 ^e^
16.	*D. canadense*	1	0	100	0
		0.5	0	100	0
		0.25	0	100	0^a^
		0.125	1.70 ± 0.01 ^x^	61.3	2.75 ± 0.16 ^d^
	*IBV (control)*	–	4.40 ± 0.09	–	5.00 ± 0.19

^x^means that the PHU number and TCID_50_ difference within the group (plant extract concentrations 0.25 and 0.125 CC_50_) was statistically significant (*P* < 0.05); if groups do not share a common letter ^a and b^ it means that the TCID_50_ (plant extract concentration 0.25 CC_50_) difference between the groups was statistically significant (*P* < 0.05); if groups do not share a common letter ^c, d and e^ it means that the TCID_50_ (plant extract concentration 0.125 CC_50_) difference between the groups was statistically significant (*P* < 0.05)

No virus plaques or CPE were detected after IBV treatment with *M. piperita*, *D. canadense* or *T. vulgaris* extracts at concentrations equivalent to between 1 and 0.25 CC_50_. The other five plant extracts exhibited a lower effect on inhibition of virus replication at the same concentrations. However, all extracts reduced the PFU number (PFU reduction rate was from 54.5 to 100.0%) and TCID_50_ (from 2.0 to 5.0 log_10_) significantly (*P* < 0.05).

Virus plaques and CPE were detected after IBV treatment with all extract concentrations equivalent to 0.125 CC_50_. All extracts decreased the PFU number (PFU reduction rate from 50.2 to 76.6%) and TCID_50_ (from 1.55 to 3.17 log_10_) significantly (*P* < 0.05). Both PFU number and TCID_50_ increased significantly after the use of *M. piperita*, *D. canadense*, *T. vulgaris* and *M. officinalis*, with extract concentrations of 0.125 CC_50_ compared to 0.25 CC_50_ (*P* < 0.05).

### Real-time RT-PCR assay

Real-time RT-PCR detected IBV RNA after treatment with all plant extracts using concentrations equivalent to 1:2 CC_50_, 1:4 CC_50_ and 1:8 CC_50_. Delta Ct values decreased significantly comparing the Ct values of 1:2 CC_50_ and 1:8 CC_50_ dilutions (*P* < 0.05, Table 4). The quantity of IBV RNA decreased significantly only after every dilution of *M. officinalis* and *H. officinalis* extracts (Table 4).

**Table 4:** Delta Ct values - comparison with the virus control by real-time RT-PCR assay

No.	Plant extract	Delta Ct values ^x^		
		1:2 CC_50_	1:4 CC_50_	1:8 CC_50_
1.	*Satureja montana*	14.06 ± 2.50^1^	3.82 ± 0.95^2^	3.32 ± 0.65^2^
5.	*Origanum vulgare*	13.28 ± 2.73^1^	7.40 ± 1.01^2^	6.36 ± 1.01^2^
6.	*Mentha piperita*	18.32 ± 2.77^1^	8.24 ± 1.06^2^	7.70 ± 1.82^2^
8.	*Melissa officinalis*	12.49 ± 2.33^1^	8.41 ± 2.22^2^	2.71 ± 0.94^3^
11.	*Thymus vulgaris*	12.65 ± 1.00^1^	11.70 ± 1.17^1^	2.66 ± 0.77^2^
12.	*Hyssopus officinalis*	10.02 ± 2.50^1^	5.33 ± 2.10^2^	2.03 ± 1.10^3^
15.	*Salvia officinalis*	8.25 ± 1.86^1^	6.65 ± 1.19^1^	1.95 ± 1.19^2^
16.	*Desmodium canadense*	16.41 ± 2.44^1^	10.58 ± 2.82^2^	6.21 ± 0.64^2^

^x^Ct of IBV was 18.50 ± 1.66; ^1, 2^ and ^3^ means that the difference within the group was statistically significant (*P* < 0.05)

### Multidimensional data analysis

**Fig. 1:**
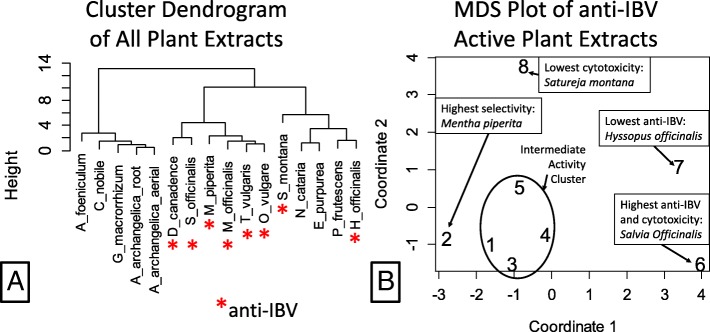
Visual representation of the experimental data. **a** Cluster dendrogram of all plant extracts. **b** Multidimensional Scaling plot of anti-IBV active plant extracts. Plant extracts: 1 – *D. canadense*, 2 – *M. piperita*, 3 – *T. vulgaris*, 4 – *M. officinalis*, 5 – *O. vulgare*, 6 – *S. officinalis*, 7 – *H. officinalis*, 8 – *S. montana*

The performed tests provided highly multidimensional data. Hierarchical clusterisation was performed using seven attributes obtained from the results of all the extracts. The seven attributes were: (1) CC_50_, (2) EC_50_, (3) SI, (4) CC_50_ dilution protecting 100% cells, (5) inhibition of cytopathic effect (CIA_100_) method 1, (6) CC_50_ dilution protecting 100% cells method 2 and (7) CC_50_ dilution protecting 100% cells method 3. Nevertheless, complete inhibition of the cytopathic effect (CIA_100_) method 4 was not used for calculations due to the fact that this experiment indicated inactivity for all tested extracts. As can be seen in Fig. 1a, three sub-clusters can be distinguished: (1) anti-IBV inactive (*A. foeniculum*, *C. nobile*, *G. macrorrhizum*, *A. archangelica* roots and aerial part), (2) anti-IBV active (*D. canadense*, *M. piperita*, *M. officinalis*, *O. vulgare, S. officinalis*, *T. vulgaris*) and (3) hybrid sub-cluster (*E. purpurea*, *S. montana*, *N. cataria*, *P. frutescens*, *H. officinalis*). *H. officinalis* and *S. montana* were clustered belonging to the hybrid sub-cluster containing anti-IBV inactive and active plant extracts. This observation can be explained by the fact that *H. officinalis* and *S. montana* exhibited the lowest cytotoxicity and the lowest anti-IBV effect among the anti-IBV active plant extracts.

The comparison of different anti-IBV active plant extracts was performed using a multidimensional scaling technique. The obtained results of anti-IBV extracts indicated highly multidimensional data (12 attributes), which cannot be visualised without additional mathematical means. The multidimensional scaling technique allows the projection of all dimensions to two dimensions, which are easily represented in the plot. In this study, 12 dimensions – (1) CC_50_, (2) EC_50_, (3) SI, (4) PFU number at dilution of 0.25, (5) PFU number at dilution of 0.125, (6) PFU reduction rate at dilution of 0.25, (7) PFU reduction rate at dilution of 0.125, (8) TCID_50_ at dilution of 0.25, (9) TCID_50_ at dilution of 0.125, (10) Delta Ct values at 1:2 CC_50_, (11) Delta Ct values at 1:4 CC_50_ and (12) Delta Ct values at 1:8 CC_50_ – were projected to two dimensionless projections (Coordinate 1 and Coordinate 2). Other attributes were not used for multidimensional scaling since they indicated similar numeric values. In the plot (Fig. 1b) four extreme points were identified: (i) *S. montana* (lowest cytotoxicity), (ii) *H. officinalis* (lowest anti-IBV activity) (iii) *S. officinalis* (highest cytotoxicity and anti-IBV activity) and (iv) *M. piperita* (highest SI). In the plot (Fig. 1b) there is a cluster of moderately active plant extracts: *O. vulgare*, *M. officinalis*, *T. vulgaris* and *D. canadense*. It should be noted that *M. piperita* extract is more similar to the moderately active cluster than other extreme points (*S. montana*, *H. officinalis*, *S. officinalis*). Interestingly, *D. canadense* is more similar to a moderately active cluster with dominating plants of family *Lamiaceae*, whereas *D. canadense* belongs to the family *Fabaceae*.

## Discussion

A wide range of traditional medicinal plants and herbs have been reported to show antiviral activities against various viruses. In this study, the anti-IBV activities were analysed of ethanol extracts from 16 medicinal plants that belong to 15 different species. In screening, the IBV *Beaudette* strain was used because it is adapted to the Vero cell culture system. A Vero cell line was used as it is one of the most common and well-established mammalian cell lines involved in assessing the effects of chemicals, toxins and other substances at the molecular level [27]. The cell line is also known to be susceptible to many viruses, including IBV, and displays CPE upon infection.

First, the cytotoxicity of plant extracts was evaluated before the possible mechanisms of virus inhibition were determined. Eight out of 16 extracts – *D. canadense, M. piperita*, *M. officinalis*, *O. vulgaris*, *T. vulgaris*, *H. officinalis*, *S. officinalis*, and *S. montana* – were chosen based on the results of the antiviral effect assay. The first seven extracts mentioned above possessed significant anti-IBV activities prior to/during infection, and only *S. montana* had an antiviral effect prior to and post-infection. All extracts, except *D. canadense*, belong to the *Lamiaceae* family, members of which have been reported in many studies as showing antiviral activity [28–30]. So far, none of these plants has been studied for antiviral activity against IBV. However, several preparations of botanicals inhibit viral replication in vitro and in vivo. Moreover, the results show that ethanol extract of *Sambucus nigra* inhibits viral replication and reduces virus titres prior to infection [24], as does *Houttuynia cordata* essential oil mixed with an aqueous solution of sodium chloride solution [25]*.* These results suggest that the effect of extracts can be associated with the direct inactivation of the envelope structures of a virus, which are necessary for adsorption to or entry into host cells, or might dissolute the IBV envelope. Thus, resistance to virucidal compounds due to mutations generated in the viral genome during replication is not likely [31].

Subsequently, the anti-IBV activities were examined of those eight plant extracts that showed the strongest antiviral activity prior to infection, using plaque reduction assay to determine the virucidal effect. Furthermore, *M. piperita, D. canadense* and *T. vulgaris* extracts exhibited the strongest viral replication inhibition, and completely stopped IBV production at 1 to 0.25 log_10_ CC_50_ concentration. Comparable results were also obtained by TCID_50_ assay. The extract of *M. officinalis* showed a slightly lower virucidal effect and at 0.25 log_10_ the CC_50_ concentration PFU number (log_10_) was 0.15 ± 0.21. It has been reported that the aqueous extract of *M. piperita* shows anti-HIV-1 activity in MT-4 cells and inhibitory activity against HIV-reverse transcriptase [32]. Moreover, hydroalcoholic and aqueous extracts of *M. piperita* and *T. vulgaris* have been shown to exert significant anti-HSV-1 and anti-HSV-2 activities in vitro. *M. piperita* essential oil has been found to be significantly effective against both HSV -1 [28] and HSV-2 when the viruses are treated with peppermint oil prior to adsorption, but not after penetration into the host cell [31]. An increase in virion density prior to its attachment to the host cells is the most likely mechanism of action of the antiviral activity of the aqueous extract of *M. piperita* [33].

The results of real-time PCR showed that this method can be successfully used to evaluate and compare the antiviral effect of plant extracts. However, it is important to note that simple or RT-PCR detects viral RNA of both an inactivated virus and an intact one. Therefore, it is not possible to assess the percentages of the nucleic acid of live and killed viruses in the sample. It is likely that the influence of the higher amounts of inactivated IBV on Delta Ct values could be significant, in some cases. It is more likely when the higher effective concentrations of plant extracts were used. RT-PCR showed that the IBV RNA quantity was statistically higher in all cases in a comparison after IBV treatment with extracts concentrations of 1:2 and 1:8 CC_50_. The RT-PCR confirmed that extracts of *M. piperita* and *D. canadense* had the highest effect on the synthesis of viral RNA.

These results were unable to reveal the most anti-IBV effective plant extract. Therefore, multidimensional data analysis was carried out using hierarchical clusterisation. It identified an anti-IBV active cluster with *M. piperita*, *T. vulgaris*, *D. canadense*, *M. officinalis*, *S. officinalis* and *O. vulgare*. Multidimensional scaling technique identified four extreme points: (i) *S. montana*, which provided the lowest cytotoxicity, (ii) *H. officinalis*, which provided the lowest anti-IBV activity, (iii) *S. officinalis*, which provided the highest cytotoxicity and anti-IBV activity, and (iv) *M. piperita*, which provided the highest SI.

In this study, the majority of plant extracts were found to have some antiviral activity and could inhibit IBV prior to and during infection. The ethanolic extracts had a mixture of compounds/fractions and it is possible that the antiviral activity of plants is decided by one or several compounds or combinations thereof [34]. Future studies are needed to detect the compounds or fractions responsible for anti-IBV activity and to investigate the mechanism of their action.

## Conclusions

Many extracts of plants acted virucidally against IBV prior to and during infection, but those of *M. piperita*, *T. vulgaris* and *D. canadense* were the most effective.

## Materials and methods

### Plants and extracts

All the plants were grown in the botanical garden of Vytautas Magnus University (Lithuania). Sixteen ethanol extracts were prepared from medicinal plants. The content of biologically active compounds is dependent on the edaphoclimatic conditions of the plant’s cultivation, the vegetation phase, the phenotype and the method of preparation of the raw material. Previous studies comparing the dried raw and fresh material of medicinal plants and different sample preparation methods have shown that drying and other conditions affect the qualitative and quantitative composition of the raw material [35, 36]. In many cases, drying is used as a standardised preparation of raw material of medicinal plants since it reduces water content and the risk of microbiological spoilage of raw material. In the present study, drying was used to collect all the samples of different plants during the intensive blooming vegetation phase for the simultaneous determination of biological activities, in consideration of the fact that different medicinal plants differ in dynamics of the accumulation of biologically active compounds, vegetation, and therefore in the harvesting of raw material [37]. The potentially antiviral plants were selected for extraction depending on the accumulated compounds in herbs, leaves and roots.

### Preparation of plant extracts

The solvent ethanol was diluted with sterile bidistilled water to 40% (vol.) concentration. Dried plant material from each plant (500 μg) was extracted with 10 ml solvent. The extraction was performed in an orbital shaker for 24 h at room temperature (20 °C). Each extract was filtrated using a paper filter and then polyvinyl difluoride membrane filter with 0.22-μm pore size. The concentration of the extracts was 50 mg/ml, with reference to the starting material. All the prepared plant extracts were stored in a refrigerator at 4 °C.

### Cell line

Vero cells (ATCC CCL-81) were provided by Dr. I. Jacevičienė from the Department of Virus Research at the National Food and Veterinary Risk Assessment Institute in Lithuania. The cells were cultivated in Dulbecco’s modified Eagle’s medium (DMEM) supplemented with 10% foetal bovine serum (FBS) at 37 °C in 5% CO_2_ incubator. Nystatin (100 units/ml) and gentamycin (50 μg/ml) were used to prevent microbial contamination.

### Virus

The Vero-adapted *Beaudette* IBV strain was used. The virus was provided by Dr. M. H. Verheije of Utrecht University in The Netherlands. The virus stocks were prepared and stored at − 80 °C in aliquots.

### Cytotoxicity assay

The cytotoxic concentration (CC_50_) was determined for each extract on Vero cells using MTT assay [38]. First, cells were seeded at a concentration of 1 × 10^4^ cells/well in a 96-well plate and grown at 37 °C for 24 h. Each extract was tested in octuplicate once. After 72 h MTT reagent (10 μl, 5 mg/ml, Sigma-Aldrich) was added and incubated for 4 h at 37 °C. Then 100 μl dimethyl sulphoxide (DMSO) (Carl Roth, Germany) was added to each well and the plates were placed on the shaker for 5 min. The absorbance of each well was measured at 620 nm in a microplate reader (Multiskan™ FC Microplate Photometer) and the percentage of cell survival was calculated. Finally, dose-response curves were plotted to enable the calculation of CC_50_ that causes lysis and death in 50% of cells.

### Screening extracts for antiviral activity

For determination of antiviral properties, one-day-old Vero cells (seeded 1 × 10^4^ cells/well) in a 96-well plate were used. The virus was used at a multiplicity of infection (MOI) of 0.05. Each extract was serially diluted twofold to 1:128 in DMEM and assessed for the ability to inhibit IBV replication using four mechanisms. Every sample of extract was tested twice in quadruplicate. In addition, controls of cells, the virus and extracts were included. An inverted microscope (Leica, Germany) was used to observe cells after all procedures.

In the first method, the virus was treated with the diluted extract for 1 h in a separate 96-well plate and then poured onto the cells. The mixtures were discarded after incubation for 1 h at 37 C in 5% CO_2_ and then the cells were washed twice with PBS. After washing, DMEM containing 2% of FBS was added. Observation by microscopy for inhibition of cytopathic effect (CPE) was performed after incubation for 72 h at 37 °C in 5% CO_2._

In the second method, the mixtures of virus and the diluted extract were poured onto the cells immediately. The mixtures were discarded after incubation for 1 h at 37 °C in 5% CO_2_ and then the cells were washed twice with PBS. After washing, DMEM containing 2% of FBS was added. Observation by microscopy for inhibition of CPE was performed after incubation for 72 h at 37 °C in 5% CO_2._

In the third method, the cells were inoculated with the virus and then treated with extract. First the cells were inoculated with the virus and incubated for 1 h at 37 °C in 5% CO_2_. Then the unadsorbed virus was discarded and the cells were washed twice with PBS. After washing, the cells were treated with the diluted extracts for 1 h at 37 °C in 5% CO_2_. After washing the cells twice with PBS, DMEM containing 2% of FBS was added. Observation by microscopy for inhibition of CPE was performed after incubation for 72 h at 37 °C in 5% CO_2._

In the fourth method, the cells were treated with extract prior to inoculation. First the cells were treated with the diluted extracts for 1 h at 37 °C in 5% CO_2_. Then the cells were washed twice with PBS and inoculated with the virus. After incubation for 1 h at 37 °C in 5% CO_2_ the cells were washed twice with PBS, and DMEM containing 2% of FBS was added. Observation by microscopy for inhibition of CPE was performed after incubation for 72 h at 37 °C in 5% CO_2._

The most promising plant extracts were selected for determination of EC_50_ and selectivity index (SI) based on the results of the antiviral effect assay.

### Determination of EC_50_ and SI

Eight out of 16 plant extracts were chosen for the determination of EC_50_ and SI using the first method. Extracts were titrated from 1 to 1:128 CC_50_ and used for virus treatment. After 72 h the MTT assay was performed as outlined above. The 50% effective concentrations (EC_50_) were calculated from the plot of percentages of cell viability against extract concentrations.

### Plaque reduction assay

Concentrations of extracts from 1 CC_50_ to 0.125 CC_50_ and 25,000 plaque-forming units (PFU) of IBV were mixed and incubated at room temperature for 1 h.

Plant extracts were diluted with DMEM to prepare four concentrations equivalent to 1 CC_50_, 0.5 CC_50_, 0.25 CC_50_, and 0.125 CC_50_, as calculated by cytotoxicity assay. These dilutions were then mixed with 25,000 plaque-forming units (PFU) of IBV and incubated at room temperature for 1 h. After incubation, a confluent monolayer of Vero cells in 6-well plates was inoculated with 1 ml of virus (MOI 0.05) and plant extract mixtures for 1 h at 37 °C in 5% CO_2_ and then discarded. The agarose 0.4% in maintenance medium was added to cells, and the plates were stored at room temperature for 15 min and incubated at 37 °C and 5% CO_2_. After 72 h the plates were microscopically examined for detection of CPE and then 0.2 ml MTT (5 mg/ml) was used for staining. Plaques were counted after incubation at 37 °C in 5% CO_2_ for 4 h. The number of plaques was expressed as log_10_ and the reduction rate was calculated as follows:

$$ \frac{\mathrm{PFU}\ \mathrm{number}\ \mathrm{of}\ \mathrm{virus}\ \mathrm{control}\hbox{-} \mathrm{PFU}\ \mathrm{number}\ \mathrm{after}\ \mathrm{the}\ \mathrm{treatment}\ \mathrm{with}\ \mathrm{plant}\ \mathrm{extract}}{\mathrm{PFU}\ \mathrm{number}\ \mathrm{of}\ \mathrm{virus}\ \mathrm{control}}\times 100\% $$

### Virus yield reduction

A virus yield reduction was evaluated by means of virus titration and real-time reverse transcriptase polymerase chain reaction (RT-PCR). The cells were inoculated as outlined above in the plaque reduction assay section. Plant extracts were diluted with DMEM to prepare four concentrations equivalent to 1 CC_50_, 0.5 CC_50_, 0.25 CC_50_, and 0.125 CC_50_ as calculated by cytotoxicity assay. These dilutions were then mixed with 25,000 plaque-forming units (PFU) of IBV and incubated at room temperature for 1 h. After incubation a confluent monolayer of Vero cells in 6-well plates was inoculated with 1 ml of virus (MOI 0.05) and plant extract mixtures for 1 h at 37 °C in 5% CO_2_, and then discarded. After inoculation DMEM containing 2% of FBS was added and the cells were incubated for 24 h. CPE of the virus was evaluated using light microscopy. After that, the plates were frozen and thawed three times and the aliquots of the virus were prepared by centrifugation for 15 min at 2000 RPM. The prepared mixtures were used for quantification of both treated and untreated virus and viral nucleic acids by means of TCID_50_ assay and quantitative real-time RT-PCR respectively.

### TCID_50_ assay

Determination of TCID_50_ of the control virus and the treated one was performed in 96-well plates. CPE was evaluated after 7 days. Virus titres were calculated using the Kärber method (Kärber, 1931).

### Real-time RT-PCR assay

Ribonucleic acid (RNA) used in the real-time RT-PCR was extracted by means of TRIzol Reagent (Thermo Fisher Scientific, USA) according to the manufacturer’s instructions. Real-time RT-PCR was performed as described by Meir [39]. Briefly, a conserved region of 336 base pairs located at nucleotide position 741–1077 of the H120 strain N gene sequence (GenBank accession no. AM260960) was used to design primers and probe for the real-time RT-PCR assay. A downstream primer IBV-f (5-ATGCTCAACCTTGTCCCTAGCA-3) located at nucleotide position 811–832, an upstream primer IBV-r (5-TCAA-ACTGCGGATCATCACGT-3) located at nucleotide position 921–941, and a TaqMan® probe IBV-TM (FAM-TTGGAAGTAGAGTGACGCCCAAACTTCA-BHQ1) located at nucleotide position 848–875 were used to amplify a 130-bp fragment. Both the primers and the probe were synthesised by Applied Biosystems, UK. The 25 μl real-time RT-PCR reaction contained 12.5 μl 2 × RT-PCR buffer mix (AgPath™ One-Step RT-PCR kit, Applied Biosystems), 1 μl 25 × RT-PCR enzyme mix (Applied Biosystems), primers to a final concentration of 400 nM, probe to a final concentration of 120 nM, 2 μl RNA template, and nuclease-free water. The reaction was carried out in StepOne™ Plus real-time PCR system (Applied Biosystems) at 45 °C for 10 min, 95 °C for 10 min, and 40 cycles of 95 °C for 15 s and 60 °C for 45 s. Amplification plots were recorded and analysed, and the threshold cycle (Ct) was determined with the Mastercycler RealPlex^2^ (Eppendorf).

The real-time RT-PCR was repeated four times, and then delta Ct values were calculated by subtracting the Ct values of virus control from Ct values of virus samples treated with plant extracts. Means and standard deviations of delta Ct values were then calculated to evaluate the effect of plant extracts on viral replication.

### Statistical and data analysis

The differences between the methods and extracts were evaluated by Fisher’s criteria and the Student’s t-test. The data were regarded as significant when *P* < 0.05. Hierarchical clusterisation and multidimensional scaling (MDS) were performed using the software R-Studio. For hierarchical clusterisation, the *Euclidean* method was used. For MDS, the *Euclidean* method was used projecting all dimensions to two dimensions.

## Data Availability

The datasets supporting the results of this document are contained within the article. Any additional data may be requested to the corresponding author.

## Abbreviations

CC_50_: 50% cytotoxic concentration
CIA_100_: Complete inhibition of cytopathic effect
CPE: Cytopathic effect
Ct: Threshold cycle
DMEM: Dulbecco’s modified Eagle’s medium
DMSO: Dimethyl sulphoxide
EC_50_: 50% effective concentrations
FBS: Foetal bovine serum
IB: Avian infectious bronchitis
IBV: Avian infectious bronchitis virus
MDS: Multidimensional scaling
MOI: Multiplicity of infection
PBS: Phosphate-buffered saline
PCR: Polymerase chain reaction
PFU: Plaque-forming unit
RNA: Ribonucleic acid
RPM: Revolutions per minute
RT-PCR: Reverse transcription polymerase chain reaction
SI: Selectivity index
TCID_50_: 50% tissue culture infectious dose
